# Detecting Foreign Bodies in a Head Laceration

**DOI:** 10.1155/2015/801676

**Published:** 2015-01-31

**Authors:** Thomas R. Fowler, Steven J. Crellin, Marna Rayl Greenberg

**Affiliations:** Department of Emergency Medicine, Lehigh Valley Hospital, USF Morsani College of Medicine, CC & I-78, Allentown, PA 18103, USA

## Abstract

Open wounds represent a potential area of medicolegal risk if foreign bodies are not identified prior to wound closure. The importance of imaging of lacerations was underscored by a recent case where a 20-year-old male collided with a friend's mouth on a trampoline sustaining a simple, superficial scalp laceration. The wound was evaluated in typical fashion including irrigation and local exploration and was prepared for closure. The friend was then evaluated and noted to have multiple extensive dental fractures. An increased index of suspicion generated further evaluation of the first patient's wound. Plain radiography obtained of the first patient's skull was noted to have bony foreign bodies consistent with teeth, which were then removed after further exploration. Superficial wounds are common and complications arising from retained foreign bodies are a potential source of substantial morbidity and consequently medical litigation. This case serves as a reminder to be vigilant and maintain a high index of suspicion regarding the potential for foreign body.

## 1. Introduction

Open wounds account for approximately 17.9% of all ED visits [1] and represent a potential area of medicolegal risk, especially when foreign bodies are not identified prior to wound closure. Complications such as dehiscence and infection from such wounds represent 14% of lawsuits and 5% of all legal settlements [2]. Standard evaluation of open wounds includes a thorough history, physical examination, wound exploration with irrigation, and occasionally debridement. Imaging should be strongly considered whenever there is concern for a potential foreign body.

## 2. Case Report

**Figure 1:**
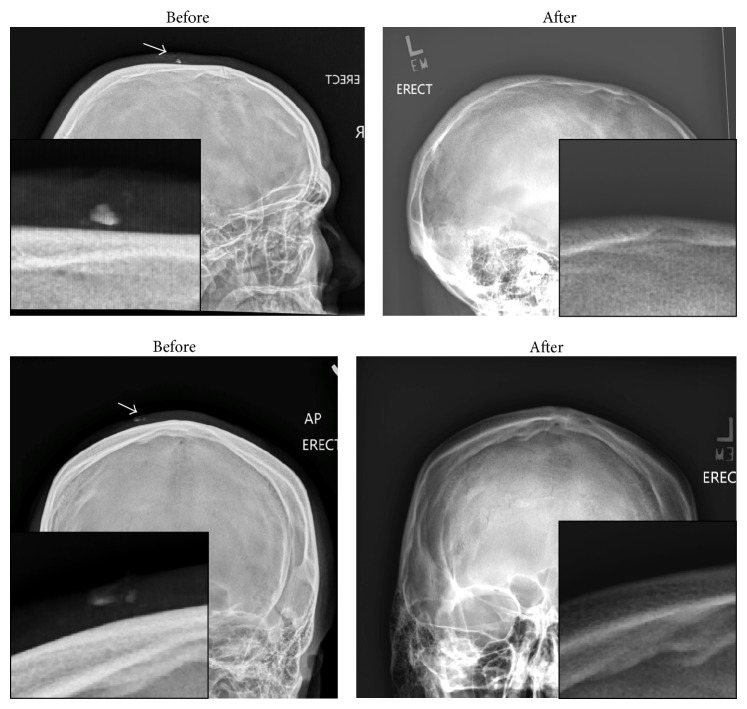
Before and after images of retained foreign bodies (fractured teeth).

The importance of imaging of lacerations was underscored by a recent case where a 20-year-old male collided with a friend's mouth on a trampoline sustaining a simple, 2.6 cm by 0.5 cm superficial scalp laceration. The wound was evaluated in typical fashion including irrigation and local exploration and was prepared for closure. The friend was then evaluated and noted to have multiple extensive dental fractures. An increased index of suspicion generated further evaluation of the first patient's wound. Plain radiography obtained of the first patient's skull was noted to have bony foreign bodies consistent with teeth, which were then removed after further exploration and without need for wound extension (Figure 1). The wound was then closed in standard fashion.

## 3. Discussion

Superficial wounds are common and complications arising from retained foreign bodies are a potential source of substantial morbidity and consequently medical litigation. In this case the foreign bodies were not readily visible even after a careful exploration. A high index of suspicion regarding the potential for foreign body is advised. Plain radiography is considered useful for viewing radiopaque foreign bodies such as metal, bone, teeth, pencil graphite, certain plastics, glass, gravel, stone, some fish spines, and wood [3]. Ultrasound may assist in detection of soft-tissue foreign bodies like glass, metals, plastics, stone, and wood with variable sensitivities because of variables like operator skill and the type of material of the foreign body [4, 5]. Other advanced imaging modalities, such as CT and MRI, may be appropriate depending on the clinical scenario. If the clinical scenario does not allow for imaging, the patient should receive explicit instructions about the possibility of retained foreign body and appropriate advice for follow-up and return.

This was a near miss event; to avoid it in the future, this case highlights the importance of constant vigilance for retained foreign bodies. It serves as a reminder to perform the appropriate radiographic investigation of simple lacerations prior to closure when the potential presence of foreign body exists.
